# Prediction of biochemical recurrence after laparoscopic radical prostatectomy

**DOI:** 10.1186/s12894-023-01350-2

**Published:** 2023-11-13

**Authors:** Tanan Bejrananda, Pitchaya Pliensiri

**Affiliations:** Division of Urology, Department of Surgery, Faculty of Medicine, Prince of Songkla University, Hatyai, Songkhla Thailand

**Keywords:** Nomogram, PSA nadir, Prediction, Biochemical recurrence, Radical prostatectomy

## Abstract

**Background:**

Radical prostatectomy (RP) has been considered primary treatment for localized prostate cancer. Biochemical recurrence (BCR) occur approximately 20–30% in five year after RP. We aim to develop a novel nomogram to predict BCR-free survival (BCRFS) and performed external validation using a validation cohort that may help clinicians to make better decision for tailoring adjuvant treatment to specific group of patients.

**Materials and methods:**

This retrospective cohort study included 370 localized and regional prostate cancer patients who underwent laparoscopic radical prostatectomy (LRP) in Songklanagarind hospital between January 2010 and December 2019, the patients were divided into two groups (primary cohort and validation cohort). BCR-free survival was created using Kaplan-Meier curve. Predictive factors for BCR were identified with univariable and multivariable analysis using Cox proportional hazards model. Predictive nomogram was created using these identified factors and developed for the prediction of biochemical recurrence free survival (BCRFS) at 1 and 5 years after LRP.

**Results:**

For primary Songklanagarind cohort, BCR was found in 105 patients (44.7%). Overall 1-year BCR-free survival was 52.8%, and 5-year BCR-free survival was 45.7% with median time to BCR of 18.1 months. Multivariable analysis identified unfavorable factor to BCRRF which are high initial serum PSA (> 20) (*p < 0.001*; HR 3.2), ISUP Gleason grade group > = 3 (*p 0.033;* HR 2.2), positive surgical margins *(p 0.046;* HR 1.5), and seminal vesicle involvement (*p < 0.001;* HR 5.2) and using for develop a novel nomogram to predict BCR. Concordance index 0.78.

**Conclusion:**

Prostate cancer patients with unfavorable factors, including high initial PSA (> 20), ISUP Gleason grade group > = 3, positive margin and extra-prostatic tumor extension are considered high risks and independent predictors of biochemical recurrence. This predictive models could potentially improve the 1 and 5-year BCR prediction after RP, according to the study’s findings and will aid medical professionals in achieving the goal of clinical prediction and creating a proper management for the localized treatment of prostate cancer underwent laparoscopic radical prostatectomy.

## Introduction

Prostate cancer is one of the most common cancers and the second leading cause of death among males. In 2023, the number of new cases and mortality from prostate cancer in the US is predicted to reach around 288,300 and 34,700, respectively [1]. One of the recommended treatments for people with prostate cancer of intermediate risk is a radical prostatectomy (RP) with concurrent pelvic lymph node dissection (PCa). Biochemical recurrence (BCR) is a side effect of radical prostatectomy that affects 20–30% of individuals [2–4]. To aid in the clinical decision-making process for following treatment, a variety of BCR prediction techniques have been created. The majority of these techniques were created using clinical and pathological characteristics, including pre-operative serum prostate-specific antigen (PSA), tumor stage, Gleason score, extracapsular extension (ECE), surgical margin (SM), and seminal vesicle involvement(SV) [5–8]. The cornerstone of post-operative follow-up is PSA measurement. Within six weeks of RP, serum PSA is anticipated to be undetectable, and patients with measurable PSA after RP are believed to have associated residual malignancies [9]. It has been demonstrated that a persistent (detectable) PSA following RP is a poor predictor of oncologic outcomes [10]. While there are existing prognostic models, it is important to note that they may not fully capture the unique characteristics and outcomes of patients undergoing LRP. Additionally, our nomogram incorporates specific variables and considerations relevant to our patient population, potentially leading to improved predictive accuracy in this specific context.

We aim to predict BCR following laparoscopic radical prostatectomy(LRP) and to guide adjuvant or salvage treatment using assessment of the prognostic power of perioperative data in predicting biochemical recurrence-free survival (BCRFS) of LRP and develop a nomogram that incorporates of common perioperative variables, to improve the accuracy of the predictive models. In this study, we note that the risk nomograms offer more precise prediction and risk stratification.

## Methods

### Patients

**Fig. 1:**
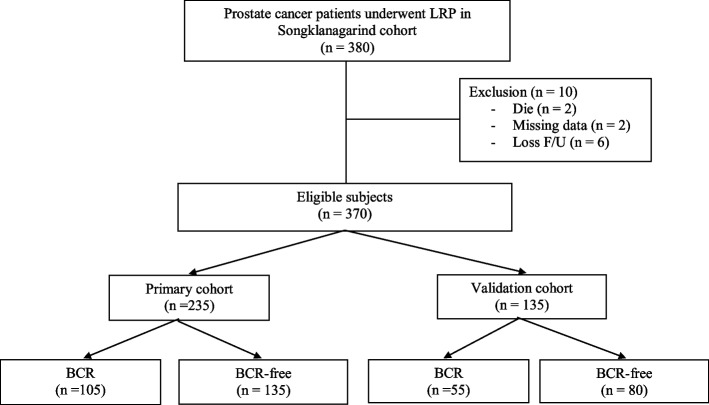
Flow chart outlining patient selection and grouping process of the study

Between January 2010 and December 2019, 370 patients who received LRP for prostate cancer were retrospectively examined. The patients were divided into two groups: 235 in the primary Songklanagarind cohort who were enrolled and 135 in the validation Songklanagarind cohort (Fig. 1). Our new nomogram was created using the primary Songklanagarind primary cohort, and validation was performed using the validation Songklanagarind cohort. After a digital rectal examination, all patients had a multicore transrectal ultrasound-guided prostate biopsy. Dedicated pathologists determined the Gleason score and the percentage of samples that were involved. All men’s pretreatment PSA levels were noted. American Joint Committee on Cancer TNM criteria was used for stagings at the time of surgery, the attending urologist determined the clinical stage. Using a contrast-enhanced kidney-ureter-bladder (KUB) and pelvic computed tomography (CT) and bone scan, all patients were preoperatively staged for metastases. Neither neoadjuvant hormone therapy nor radiation therapy was administered to the patients.

The following patients met the exclusion criteria: (1) those who had undergone transurethral resection of the prostate; (2) those whose pathology findings did not indicate prostatic cancer; and (3) those with insufficient follow-up information. Every 3 months for the first two years, semi-annually for the third and fourth years, and then annually after that, follow-ups were conducted.

In the first two follow-ups following RP without adjuvant androgen deprivation therapy or radiation, the PSA nadir was defined as the lowest level of serum PSA. BCR was defined as a post-operative PSA value > 0.20 ng/mL in two successive readings, and the recurrence date was given as the day on which a PSA value > 0.20 ng/mL was recorded for the first time. For patients who did not have BCR, BCRFS was computed from the RP date to the date of verified BCR or the date of the most recent follow-up. Age at RP, body mass index (BMI), pre-operative prostatic specific antigen(PSA), Gleason score, present of surgical margin (SM), extracapsular extension(ECE), seminal vesicle involvement (SVI), and lymphovascular invasion.

(LVI) were additional clinical and pathological details that were gathered for each patient. The analysis collected from patients with a follow-up period longer than 12 months.

### Statistical analysis

Survival analyses for biochemical recurrence free were performed using the Kaplan‒Meier method for patients who survived 1 and 5 years. We used the log-rank test to compare between groups. We employed both univariable and multivariable logistic regression analyses to identify potential predictors. Variables that showed statistical significance (*P* < 0.05) in the univariable logistic analysis of the training set were subsequently included in the multivariable logistic regression analysis. The aim was to identify independent predictors for BCR. Those predictors that demonstrated statistical significance (*P* < 0.05) in the multivariable logistic regression analysis were included in constructing the nomogram. To assess the goodness of fit, we applied the Hosmer and Lemeshow test, where a *P*-value greater than 0.05 indicated a good fit. Additionally, we calculated the odds ratio (OR) and 95% confidence interval (95% CI) for the identified predictors. Univariable and multivariable Cox models after LRP were used to identify predictors of BCR and assess time trends in BCR. Using the significant variables, we developed a nomogram predicting BCRFS at 1 and 5 years after LRP. For internal and external validation of the nomogram, we used a bootstrap using 500 resamples to assess the discrimination and calibration. It was determined whether there was discrimination using the concordance index (c-index). The calibration curve was created to evaluate the calibration and visually depict the relationship between the projected probability of the BCR and the actual observed events. Discrimination was quantified by calculating the area under the receiver-operating characteristic curve (AUC). To evaluate the external validity of the prediction model, the validation cohort (as test cohort) was used to obtain ROC curves and AUCs from the predicted values by fitting the model created by the primary Songklanagarind cohort (as training cohort). The precision of the predicted probability when fitting the prediction model to the validation data was verified using a calibration plot. R was used to conduct statistical analysis (version 4.0.1, R Foundation for Statistical Computing, Vienna, Austria). All statistical tests were two-sided, and *P* < 0.05 was considered statistically significant.

## Results

### Patients characteristics

As a result, 235 patients in the primary Songklanagarind cohort and 135 patients in the validation cohort fulfilled the criteria of this study. The median follow-up period was 40 months (IQR), 19–60 months). The demographic and clinical variables of the patients who were included in this study overall are shown in Table 1. The median age of primary Songklanagarind patients was 68.4 years (IQR, 64–73 years) with median BMI of 24.4 kg/m2 (IQR, 22.6– 26.2 kg/m2). The median value of pre-operative PSA was 13.6 ng/mL (IQR, 9.3– 24.8 ng/mL) and was divided into 2 groups: primary Songklanagarind and validation cohorts. (Table 1)

**Table 1:** Demographic data of all prostate cancer patient who underwent laparoscopic radical prostatectomy included in the analysis for primary cohort and validation cohort

Variables	All	Primary cohort	Validation	*p*-value
**Total**	370	235	135	
**Age (yrs)**				0.422
** Mean (IQR)**	67.7 (63–72)	68.4 (64–73)	67 (62–72)	
**BMI (Kg/m**^**2**^**)**				0.365
** Median (IQR)**	24.3 (22.3,26.4)	24.4 (22.6,26.2)	24.2 (21.2,26.4)	
**Unknown**	7	5	2	
**Blood loss (mL)**				0.542
** Median (IQR)**	520 (280,820)	500 (300,800)	540 (280,820)	
**Prostate size**				0.416
** Median (IQR)**	46 (32.52)	42 (33.5,53.5)	50 (32,56)	
**Unknown**	6	4	2	
**PSA group (ng/mL)**				0.128
** <= 20**	254 (68.6)	153 (75.1)	101 (74.8)	
** >20**	116 (31.4)	82 (34.9)	34 (25.2)	
**Pre operative MRI (PIRSADs)**				0.419
2	5 (1.4)	4 (1.7)	1 (0.7)	
3	13 (3.5)	9 (3.8)	4 (3.0)	
4	137 (37)	87 (37.0)	50 (37.0)	
5	215 (58.1)	135 (57.5)	80 (59.3)	
**PSA preoperative**				0.6
** Median (IQR)**	13.5 (9.0,24.8)	13.6 (9.3,24.8)	13.5 (9.0,25.0)	
**Gleason**				0.365
6	81 (21.9)	61 (26)	20 (14.8)	
7	217 (58.7)	127 (54)	90 (66.7)	
8	30 (8.1)	20 (8.5)	10 (7.4)	
9	36 (9.7)	26 (11.1)	10 (7.4)	
10	6 (1.6)	1 (0.4)	5 (3.7)	
**Gleason group**				0.225
6	91 (25.6)	61 (26)	30 (22.2)	
7	197 (53.2)	127 (54)	70 (51.9)	
>=8	82 (22.2)	47 (20)	35 (25.9)	
**Grade group**				0.437
1	109 (29.4)	61 (26)	48 (35.6)	
2	128 (34.6)	78 (33.2)	50 (37.0)	
3	67 (18.2)	49 (20.9)	18 (13.3)	
4	29 (7.8)	20 (8.5)	9 (6.7)	
5	37 (10)	27 (11.5)	10 (7.4)	
**Margin**				0.286
Negative	233 (63.0)	161 (68.5)	72 (53.3)	
Positive	137 (37.0)	74 (31.5)	63 (46.7)	
**LVI**				0.263
Negative	285 (77.0)	180 (76.6)	105 (77.7)	
Positive	85 (22.3)	55 (23.4)	30 (22.3)	
**Total lymph node**				0.492
** Median (IQR)**	4 (2,7)	4 (2,6)	4 (2,7)	
**T**				0.476
1	103 (27.8)	63 (26.8)	40 (29.6)	
2	180 (48.6)	115 (48.9)	65 (48.1)	
3a	37 (10)	23 (9.8)	14 (10.3)	
3b	50 (13.5)	34 (14.5)	16 (11.8)	
**BCR rate**	165 (44.6)	105 (44.7)	60 (44.4)	0.324
**Time to BCR**				0.422
Median (IQR)	17.6 (3.6,41.2)	18.1 (3.8,41.1)	17.2 (3.6,40.0)	

### Biochemical recurrence (BCR)

**Fig. 2:**
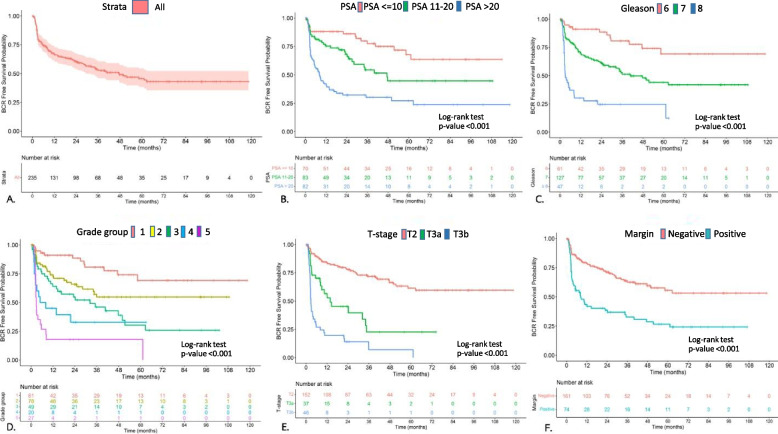
**(A-F).** BCR-free survival probability; **A**: Overall, **B**: PSA level, **C**: Gleason risk group, **D**: Gleason grade group, **E**: T stage and **F**: Surgical margin

Primary Songklanagarind cohort, 105 (44.6%) patients developed BCR during follow-up in our cohort. All patients who experienced biochemical recurrence (BCR) received radiotherapy treatment without adjuvant treatment. The 1-year and 5-year BCRFS was 52.8% and 45.7%. Median time to BCR 18.1 months and BCR-free survival probability of our cohort (Fig. 2A) and according to the PSA level, Gleason score, Gleason group, Gleason grade group, T stage and surgical margin. Kaplan-Meier curves were stratified by PSA level (< 10,10–20, ≥ 20 ng/mL) [Fig. 2B], Gleason risk group (6,7, 8–10) (Fig. 2C), Gleason grade group (1,2,3,4,5) [Fig. 2D], T stage ( < = 2, 3a, 3b) (Fig. 2E), and surgical margin (Fig. 2F) had significantly shorter BCRFS (*P* < 0.001). (Figure 2B and F).

### Development and evaluation of the novel nomogram

Pre-operative PSA was assessed using a cut-off of 20 ng/mL, Gleason grade group, SM, LVI, pT stage, and pN stage in a univariable Cox proportional hazards regression model with the results displayed in Table 2. Every predictor, with the exception of Gleason score and LVI, was statistically substantially correlated with BCR following RP (*P* < 0.01).

**Table 2:** Predictive factors - Univariable analysis and multivariable analysis

Variables	Univariable analysis				Multivariable analysis		
	HR	95% Cl	*P* value		HR	95% Cl	*P* value
Initial PSA (ng/mL)							
<=20	Ref				Ref		
> 20	4.4	2.6–7.7	< 0.001		3.2	1.8–5.8	< 0.001
Gleason score							
6	Ref						
7	2.7	1.5–5.1	0.002		2.2	1.2–3.3	< 0.001
≥ 8	8.4	4.3–16.3	< 0.001		5.4	2.6–6.8	< 0.001
Gleason grade group							
1	Ref						
2	2.2	1.1–4.2	0.025		1.5	0.7–2.9	0.29
>=3	5.1	2.8–9.6	< 0.001		2.2	1.1–4.4	0.022
Margin status							
Negative	Ref				Ref		
Positive	2.6	1.8–3.9	< 0.001		1.5	1.0-2.3	0.046
LVI status							
Negative	Ref				Ref		
Positive	2.7	1.8–4.1	< 0.001		1.0	0.6–1.6	0.841
pT stage							
2	Ref				Ref		
3a	3.2	1.9–5.4	< 0.001		2.8	1.6–4.8	< 0.001
3b	8.4	5.3–13.3	< 0.001		5.2	3.1–8.6	< 0.001
pN stage							
0	Ref				Ref		0.028
1	4.8	2.6–9.1	< 0.001		3.1	1.7–5.8	

### Predictive nomogram

**Fig. 3:**
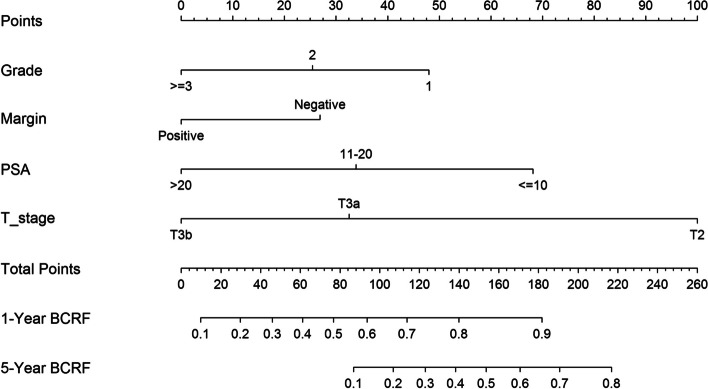
Nomogram for BCR prediction calculation for 1 and 5-year BCRFS

**Fig. 4:**
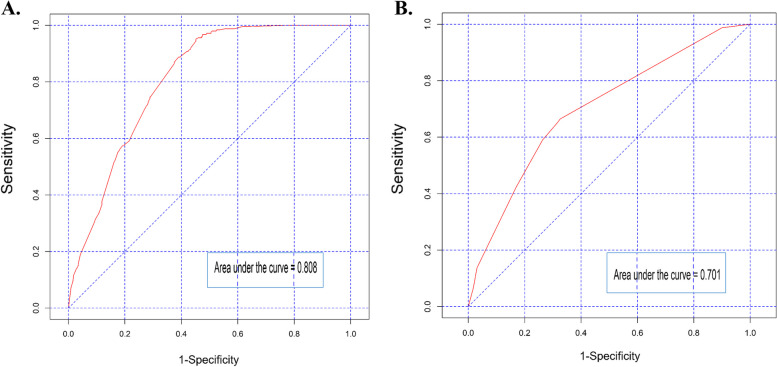
Receiver operating characteristic curve analyses of predictors for biochemical recurrence free survival with area under the curve (AUC) of 0.808 and 0.701, respectively: (**A**) primary cohort, (**B**) validation cohort

**Fig. 5:**
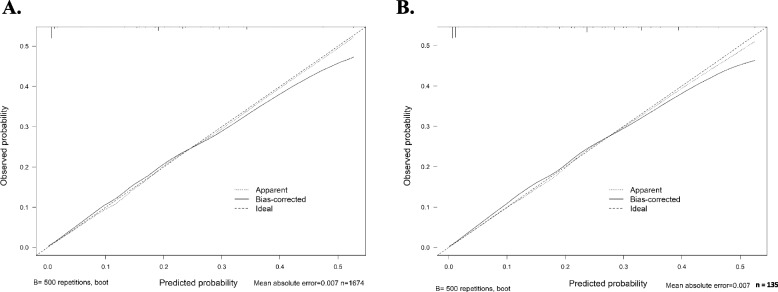
The calibration curve illustrates how the predictions from the nomogram compare with actual outcomes for the 235 patients (primary cohort) and 135 patients. The concordance index was 0.78 and 0.72, respectively.; (**A**) primary cohort, (**B**) validation cohort

Finally, PSA preoperative, Gleason grade group, SM, and pT stage, were independent predictors of BCR in multivariable Cox regression analysis (*P* < 0.05). These factors were included in a nomogram (Fig. 3), which predicted BCRFS at 1 and 5 years after RP. Figure 4 shows the ROC curve for primary Songklanagarind cohort and validation cohorts, with AUCs of 0.808 and 0.701, respectively (Fig. 4), and the calibration curve (Fig. 5) illustrates how the predictions from the nomogram compare with actual outcomes produced a c-index of 0.78 (95% confidence interval [CI], 0.71–0.81). The concordance index for this nomogram was 0.78 (primary cohort) and 0.72 (validation cohort) based on the fitted multivariable Cox model.

## Discussion

In the current study, we suggested a new nomogram that demonstrated good accuracy of BCR prediction for patients after LRP. The most crucial post-operative parameter to be evaluated is PSA, which is anticipated to be undetectable in 6 weeks following RP [9]. A high probability of local recurrence or metastasis is indicated by an elevated PSA level following RP [10]. The patient is given the status of BCR [11], which was a signal of cancer progression at a visual undetectable level, if the post-operative PSA level reaches 0.2 ng/mL. Numerous studies have been done on the connection between PSA nadir and BCR following RP. According to a retrospective analysis, men with PSA > = 0.01 ng/mL after RP had a lower chance of developing BCRFS after five years, dropping from 92.4 to 56.8% [12]. PSA persistence after RP was linked to higher BCR and overall mortality in a research by Matsumoto et al. [13], which included 582 patients. These outcomes are consistent with the findings of the current investigation. In comparison to the present analysis, where BCR was detected in 105 patients, several studies have described patients who underwent prostatectomy with a 5-year BCR-free survival rate ranging from 74 to 87% and a median PSA recurrence time of 2.6 years, which also higher compared to our study [14, 15]. Moreover, multivariable analysis identified unfavorable factor to BCRFS which are high initial serum PSA (> 20), ISUP Gleason grade group > = 3, positive margin status and seminal vesicle invasion using for develop a nomogram to predict BCR. Consistent with previous literature, patients with pathologically organ-confined cancer exhibited consistently low hazard rates over the follow-up period, emphasizing the significance of extended surveillance and timely intervention in individuals who undergo radical prostatectomy [16]. Furthermore, positive surgical margins (PSM) have been shown to have a detrimental impact on postoperative functional outcomes (PFP) following radical prostatectomy (RP), emphasizing the need to minimize PSM rates for improved cancer control outcomes, despite the growing prevalence of organ-confined disease resulting from enhanced screening strategies [17]. Similar conclusions have been reported regarding the nomogram in previous studies [18, 19].

Previously, Cooperberg et al. [7] developed the post-operative CAPRA-S score based on pre-operative PSA, Gleason score, seminal vesicle involvement, lymphonodal extension, surgical margin invasion, and Extracapsular extension. We also acknowledge the existence of the nomogram available on the Memorial Sloan Kettering Cancer Center (MSKCC) website, which has been widely used as a valuable tool in predicting BCR [20]. While the MSKCC nomogram has demonstrated its utility and validity in the field, our novel nomogram expands upon the existing models in several key ways. First and foremost, our nomogram incorporates a focus group of the laparoscopic approach, which allows for enhanced generalizability of the predictive model, which was not present in the MSKCC nomogram. By including these updated factors, our nomogram aims to provide an improved and more accurate prediction of BCR after laparoscopic radical prostatectomy. Through these comparative analyses, we have demonstrated that our nomogram exhibits superior predictive performance when applied to our specific patient cohort. This enhanced performance can be attributed to the incorporation of additional relevant variables and a refined model development process.

Our study revealed a significantly higher positive surgical margin (PSM) rate of 31.5% compared to previous findings. According to the previous study by Guillonneau et al. [21] The PSM rates of 6.9%, 18.6%, 30%, and 34% for for pT2a, pT2b, pT3a, and pT3b stages, respectively., our study had a lower proportion of T3 patients at 24.3%. This discrepancy may contribute to the higher overall PSM rate, as T3 tumors typically have increased extracapsular extension and involvement of surgical margins. However, additional analysis is needed to explore other factors such as surgical technique, surgeon expertise, patient characteristics, tumor aggressiveness, and pathological assessment methods. Understanding these factors can inform improvements in procedures, patient selection, and postoperative care to reduce positive surgical margins and enhance outcomes for laparoscopic radical prostatectomy patients.

Due to variations in prostate cancer phenotypes across people in Asian and Western nations, results from Asian countries regarding the prediction of BCR are currently missing [22]. There are many risk factor and its impact on the BCR rate following surgery are still challenging for surgeons to predict. To advance the present understanding of BCR after LRP, we used retrospective cohort data from Songklanagarind hospital in this study.

We created a simple model with comparisons for the PSA cut-off at 20 ng/mL level, Gleason grade group, SM, and pathologic T stage in order to confirm the incremental predictive value of the combination of independent clinical indicators. Our newly developed nomogram has the potential to serve as a valuable tool for predicting biochemical recurrence-free survival (BCRFS) and aiding in the decision-making process for adjuvant treatments. Specifically, when the nomogram indicates a high probability of biochemical recurrence (BCR) or a low score in BCRFS prediction, it suggests the consideration of early adjuvant radiotherapy. Conversely, when the nomogram indicates a low probability of BCR, it may provide the opportunity to omit adjuvant radiotherapy and avoid potential side effects associated with such treatment.

The limitations of this study. First, because this was a retrospective study and the number of patient was much smaller than in other studies, this study has a number of drawbacks. Second, the newly created nomogram, which predicts BCRFS following LRP with good accuracy, incorporated various widely used factors, including PSA, Gleason grade group, and pathologic T stage. Specifically, we acknowledge that all patients underwent transrectal ultrasound-guided prostate biopsy instead of MRI-guided prostate biopsy, which could potentially impact the validity of the nomogram. Moreover, validation cohort was used to confirm accuracy of our nomogram. We emphasize that future studies should aim to externally validate the nomogram in different clinical settings to ensure its broader applicability.

While we agree that MRI-guided biopsy has advantages in terms of accuracy, availability, and target localization, it is important to note that at the time of our study, transrectal ultrasound-guided biopsy was the standard practice in our institution. Nonetheless, we recognize the potential limitations associated with this choice and will include a discussion on the impact of biopsy technique in the revised version of our article. To enhance accuracy, we will incorporate multiple evaluation methods, including PSA levels and other biomarkers or imaging techniques. Implementing these measures will provide a more comprehensive analysis of biochemical recurrence following laparoscopic radical prostatectomy.

The practical implementation of the nomogram in clinical practice to guide adjuvant or salvage treatment is an essential consideration for its real-world applicability. Clinicians interested in utilizing the nomogram as a decision-making tool would benefit from additional clarification on its implementation. Firstly, it is important to provide guidance on how to use the nomogram in a clinical setting. This includes outlining the specific variables and inputs required for accurate predictions. Clear instructions on data collection, such as the recommended imaging modalities or laboratory tests, would facilitate the practical application of the nomogram. Furthermore, discussing the interpretation of the nomogram’s results is crucial. Clinicians need to understand the threshold or cutoff values associated with different treatment recommendations. Guidance on the level of confidence or uncertainty in the predictions would also assist clinicians in evaluating the nomogram’s reliability. In addition to implementation and interpretation, the authors should consider discussing the potential benefits and limitations of integrating the nomogram into clinical practice. Highlighting the advantages, such as its ability to provide individualized risk assessment and guide treatment decisions, would emphasize its utility. However, it is equally important to address any limitations, such as the nomogram’s reliance on specific patient populations or potential biases in the development of the model.

## **Conclusion**

Based on routine variables discovered in prostate cancer patients with unfavorable factors, such as high initial PSA (> 20), ISUP Gleason grade group > = 3, positive margin, and higher pathologic T stage, this predictive models could potentially improve the 1 and 5-year BCR prediction after RP, according to the study’s findings and will aid medical professionals in achieving the goal of clinical prediction and creating a proper management for the localized treatment of prostate cancer underwent laparoscopic radical prostatectomy.

## Data Availability

The datasets used and analyzed during the current study are available from the corresponding author upon reasonable request.
